# HMGB1 in Pediatric COVID-19 Infection and MIS-C: A Pilot Study

**DOI:** 10.3389/fped.2022.868269

**Published:** 2022-04-26

**Authors:** Laura Petrarca, Valeria Manganelli, Raffaella Nenna, Antonella Frassanito, Shira Ben David, Enrica Mancino, Tina Garofalo, Maurizio Sorice, Roberta Misasi, Fabio Midulla

**Affiliations:** ^1^Maternal Infantile and Urological Sciences Department, Sapienza University of Rome, Rome, Italy; ^2^Translational and Precision Medicine Department, Sapienza University of Rome, Rome, Italy; ^3^Department of Experimental Medicine, Sapienza University of Rome, Rome, Italy; ^4^Policlinico Umberto I Hospital, Rome, Italy

**Keywords:** MIS-C multisystem inflammatory syndrome in children, COVID–19, HMGB1 (high mobility group box 1), SARS–CoV2, infection - immunology

## Abstract

**Objective:**

Since the beginning of the coronavirus disease 2019 (COVID-19) pandemic, a novel syndrome known as a multisystem inflammatory syndrome in children (MIS-C) was reported in previously healthy children. A possible pro-inflammatory molecule, high-mobility group box 1 (HMGB1), may be assumed to play an important role in the pathogenesis and clinical presentation of MIS-C. We described the clinical picture of patients with MIS-C and we also aimed to test and compare HMGB1 serum levels of MIS-C patients with those of patients with previous SARS-CoV2 infection and healthy children.

**Study design:**

We determined HMGB1 levels by Western blot in 46 patients and divided them into three groups, namely, five patients with MIS-C (median age: 8.36 years), 20 children with a history of SARS-CoV-2 infection (median age: 10.45 years), and 21 healthy children (controls) (median age: 4.84 years), without evidence of respiratory infection in the last 3 months.

**Results:**

The median level of HMGB1 in the serum of five patients with MIS-C was found to be significantly higher compared with both patients with a recent history of COVID-19 (1,151.38 vs. 545.90 densitometric units (DU), *p* = 0.001) and control (1,151.38 vs. 320.33 DU, *p* = 0.001) groups. The HMGB1 level in MIS-C patients with coronary involvement had a slightly higher value with respect to patients without coronary dilatation (1,225.36 vs. 1,030.49 DU, *p* = 0.248). In two of the five children with MIS-C that performed a follow-up, the HMGB1 value decreased to levels that were superimposable to the ones of the control group.

**Conclusion:**

The significantly high level of HMGB1 protein found in the serum of COVID-19 and patients with MIS-C supports its involvement in inflammatory manifestations, suggesting HMGB1 as a potential biomarker and therapeutic target in patients with severe illness.

## Introduction

The spread of severe acute respiratory syndrome coronavirus 2 (SARS-CoV2) at the beginning of 2020 has raised many doubts and uncertainties as to whether children could be affected by more severe forms of the coronavirus disease 2019 (COVID-19). As time passed, it became clear that pediatric patients are spared severe forms of COVID-19. The reason is not completely clarified, but there are studies that age-dependent expression of some host proteins, such as angiotensin-converting enzyme 2 (ACE2) and transmembrane serine protease 2 (TMPRSS2), could be responsible for less severe forms of COVID-19 in children (1). In addition, some age-dependent immune-related factors can play a role, a stronger innate immune response especially of the nasal mucosa, that can promptly control the virus (2).

Despite the less severity of COVID-19 in children, rare cases of a syndrome, characterized by multiorgan involvement and systemic inflammation, called multisystem inflammatory syndrome (MIS-C), have been described, with clinical features similar to Kawasaki disease (KD) (3). Most studies report a temporal relationship of 4–6 weeks between a previous SARS-CoV2 infection and MIS-C (4, 5).

Clinically, when it affects children, MIS-C may be present with a state of hyperinflammation characterized by fever and various clinical manifestations, including gastrointestinal symptoms (e.g., abdominal pain, vomiting, and diarrhea), neck pain, mucocutaneous rash, conjunctivitis, or fatigue and in severe cases with hypotension and shock, as defined by Centers for Disease Control and Prevention (CDC) (6).

Severe COVID-19 infection and death in adults and elderly is often caused by uncontrolled immune activation and hyperinflammation (7), and among a large number of known inflammatory proteins, high levels of high-mobility group box 1 (HMGB1) have been correlated with severe clinical manifestation (8), acute lung injury, and fatal outcome (9).

High-mobility group box 1 protein is an intracellular, non-histonic DNA-binding protein involved in DNA transcription and repair. HMGB1 protein is also found extracellular, either released passively from damaged and apoptotic cells or actively from activated immune cells (10). HMGB1 is an alarmin, whose circulating levels may be elevated during chronic inflammation, autoimmune diseases, or after surgical/anesthesia trauma (11). It is a damage-associated molecular pattern (DAMP) molecule that acts as a pro-inflammatory signal by binding and activating toll-like receptors (TLRs) as well as by binding to a receptor for advanced glycosylation end products (RAGE), enhancing innate immune response, cytokine production, and inflammation.

There are numerous publications on the functional role of HMGB1/RAGE and TLRs in enhancing the nuclear factor kappa B (NF-κB) signaling pathway and mitogen-activated protein kinases (MAPK) (10, 12), regulating the inflammatory immune response. Furthermore, recent observations demonstrate a close association between high mobility group box 1 (HMGB1), chronic inflammation, and autoimmune diseases (13–15).

Many chronic inflammatory diseases are characterized by increased levels of circulating HMGB1, perhaps of importance for the increased risk of severe outcomes in COVID-19 patients with inflammatory comorbidities. Excessive amounts of extracellular HMGB1 release cause tissue damage and organ dysfunction (16). In fact, HMGB1/RAGE/NF-κB was found to be highly expressed in the coronary arterial endothelium of children with KD, during the acute phase (17), suggesting a role in coronary inflammation and dilatation. In addition, high levels of HMGB1 in patients with KD have demonstrated resistance to intravenous immunoglobulin (IVIG) therapy (17).

Addressing the characteristic hyperinflammatory state in MIS-C, in this study, we decided to investigate the role of HMGB1 in this severe post-SARS-CoV2 clinical manifestation.

The aims of our study were as follows:

1. To describe the clinical picture of patients with MIS-C hospitalized in the Pediatric Department of Policlinico Umberto I Hospital, during the period November 2020–March 2021.
2. To test and compare patients with MIS-C HMGB1 serum levels to those of patients with a recent history of COVID-19 and healthy children.

## Materials and Methods

### Patients

Our study included the following three groups of patients:

1. Patients who fulfilled the MIS-C definitions (18, 19): age <21 years presenting with fever, laboratory evidence of inflammation, multisystemic (>2) organ involvement and severe clinical illness requiring hospitalization, no alternative plausible diagnosis with evidence of current or recent infection of SARS-CoV-2 by RT-PCR, and positive results of anti-SARS CoV-2 antibodies detected by chemiluminescence immunoassay (CLIA). In the MIS-C group, we collected the blood sample at admission, before the start of the IVIG/steroid therapy. MIS-C occurs 4–6 weeks after the SARS-CoV2 infection. Clinical and laboratory data were obtained from clinical charts. Only one patient required intensive care unit admission for severe hypotension and initial cardiac failure.
2. Twenty children (median age: 10.46, age-range: 4.58–14.58 years) with a history of SARS-CoV-2 infection, enrolled at least 1 month after the clinical recovery and/or SARS-CoV-2 swab negativization (median: 40.5 days). For each child, we recorded the presence of clinical symptoms during the infection.
3. Twenty-one healthy children (median age: 4.84, age-range: 0.35–17.16 years), 15 enrolled in the pre-pandemic period (October 2014–March 2015) and 6 patients' siblings with SARS-CoV2 that tested negative for the swab for anti-SARS-CoV2 antibodies and with no history of infection in the previous 2 months.

None of the patients were subjected to SARS-CoV2 vaccination.

### Ethics Approval

The research and ethics committee of the Policlinico Umberto I Hospital approved this study (no. 0399/2021). This study has been carried out in accordance with the Declaration of Helsinki.

### Determination of Serum HMGB1 Levels

To avoid the possibility that serum components capable of binding to HMGB1 could interfere with its detection, we tested HMGB1 by Western blot, instead of ELISA (14, 20, 21).

Western blot to evaluate the serum level of HMGB1 in children was performed at the Experimental Medicine Department, “Sapienza” University of Rome.

In brief, sera (3 μl) obtained from each subject under test were diluted with 72 μl radioimmunoprecipitation assay (RIPA) buffer and heated at 95°C for 2 min in sodium dodecyl sulfate-(SDS) loading buffer (22). For immunodetection, the proteins were separated by 12.5% SDS-polyacrylamide gel electrophoresis (SDS-PAGE) and transferred onto polyvinylidene fluoride (PVDF) transfer membranes (Amersham Biosciences, Piscataway, NJ, USA). The membrane was blocked at room temperature for 1 h with Tris-buffered saline that contains 25 mM Tris–HCl, 150 mM NaCl, pH 7.4, and 0.05% Tween-20 (TBS-T) with 3% bovine serum albumin (BSA).

The membranes were incubated with anti-HMGB1 polyclonal antibody (1:1,000; Abcam, Cambridge, UK). The primary antibody was applied for 2 h at room temperature, followed by four 15-min washes with TBS-T.

The secondary antibody was horseradish peroxidase-conjugated anti-rabbit (1:10,000, Sigma-Aldrich, Milan, Italy) at room temperature. After washing, proteins were detected using ECL reagents (Amersham Biosciences). HMGB1 standard (rHMGB1, Sigma-Aldrich) sample was prepared by adding SDS buffer and was included in each blot as an internal control. Densitometric analysis was performed using the ImageJ software (National Institutes of Health, Bethesda, MD, USA).

### Statistical Analysis

All the statistical procedures were performed using GraphPad Prism Software Inc. (San Diego, CA, USA) and SPSS (IBM, v. 25). Normally distributed variables were summarized using the mean ± standard deviation (SD), and not normally distributed variables were by the median and range. Differences between numerical variables were tested using the Wilcoxon test or the Kruskal-Wallis test. *P*-values < 0.05 were considered significant.

## Results

### Patients With MIS-C

During the study period (November 2020–March 2021), five patients (3 males, mean age: 8.36 years, age range: 1.59–10.13 years) fulfilled the MIS-C diagnostic criteria (6). They were all admitted for persistent fever and pharyngitis, and were not responsive to antibiotic treatment. All the patients presented a diffuse mucocutaneous rash, and three patients also presented enlarged cervical lymph nodes (diameter higher than 1 cm). Three patients had cardiac involvement with coronary dilatation and one of them also had a reduction of the ejection fraction up to 40% with severe hypotension that required inotropic treatment and pediatric intensive care unit admission. All patients had an involvement of at least two different systems and had evidence of positive IgG anti-SARS-CoV2 antibodies (Table 1). Laboratory tests showed increased C-RP levels in four patients, mild hypertransaminasemia in three patients, lymphopenia in three patients, and thrombocytopenia in two patients (Table 2). None of them reported a recent history of symptomatic SARS-CoV2 infection.

**Table 1:** Clinical characteristics of patients with multisystem inflammatory syndrome in children (MIS-C).

	**MIS-C patients (*n* = 5)**
Male gender *n* (%)	3 (60)
Median Age (range)	8.36 (1.58–10.13) years
Clinical Presentation:	
• Fever (>38°C) • Gastrointestinal symptoms • Skin rash • Lymphoadenopathy • Cardiac involvement with coronary dilation	5 (100) 4 (80) 5 (100) 2 (40) 3 (60)
Hypotension	1 (20)
Positive SARS-CoV2 swab (by RT - PCR)	1 (20)
Positive IgG anti-SARS-CoV2 (anti spike protein)	5 (100)
ICU admission	1 (20)
Treatment	
• IVIG • IV steroids • Aspirin • Heparin • Azytromicin	4 (80) 2 (40) 3 (60) 1 (20) 1 (20)

**Table 2:** Laboratory characteristics of patients with MIS-C.

	**MIS-C patients (*n* = 5)**
Total White blood cell count (n/mm^3^) median (range)	7,430 (3,600–10,720)
Lymphocyte (n/mm^3^) median (range)	690 (330–1,940)
Neutrophils (n/mm^3^) median (range)	5,770 (1,290–9,410)
Hemoglobin (g/dL) median (range)	11.5 (10.9–12.7)
Platelets (n/mm^3^) median (range)	1,42,000 (58,000–2,43,000)
C-RP (mg/dL) median (range)	10.31 (0.8–28.38)
AST (U/L) median (range)	73 (26–98)
ALT (U/L) median (range)	30 (28–109)
Albumin (g/dL) median (range)	3.6 (2.7–4)
Sodium (mmol/L) median (range)	131 (130–135)
LDH (U/L) median (range)	494 (185–1453)
D-dimer (μg/L) median (range)	4,300 (3,965–4,518)
Fibrinogen (g/L)	518 (3.04–7.58)
Ferritin (μg/mL) median (range)	558 (192–1383)

All patients but one were treated with a single dose of 2 mg/kg IVIG followed by defervescence and improvement of the clinical conditions. In one patient, IVIG was associated with a bolus of 500 mg of methylprednisolone, followed by 1 mg/kg of prednisolone gradually reduced after 21 days due to severe cardiac involvement. One patient was treated with IV steroids only (7 days of IV methylprednisolone 2 mg/kg, followed by 1 mg/kg of prednisolone gradually reduced after 21 days) (Table 1).

### Serum Levels of HMGB1

**Figure 1:**
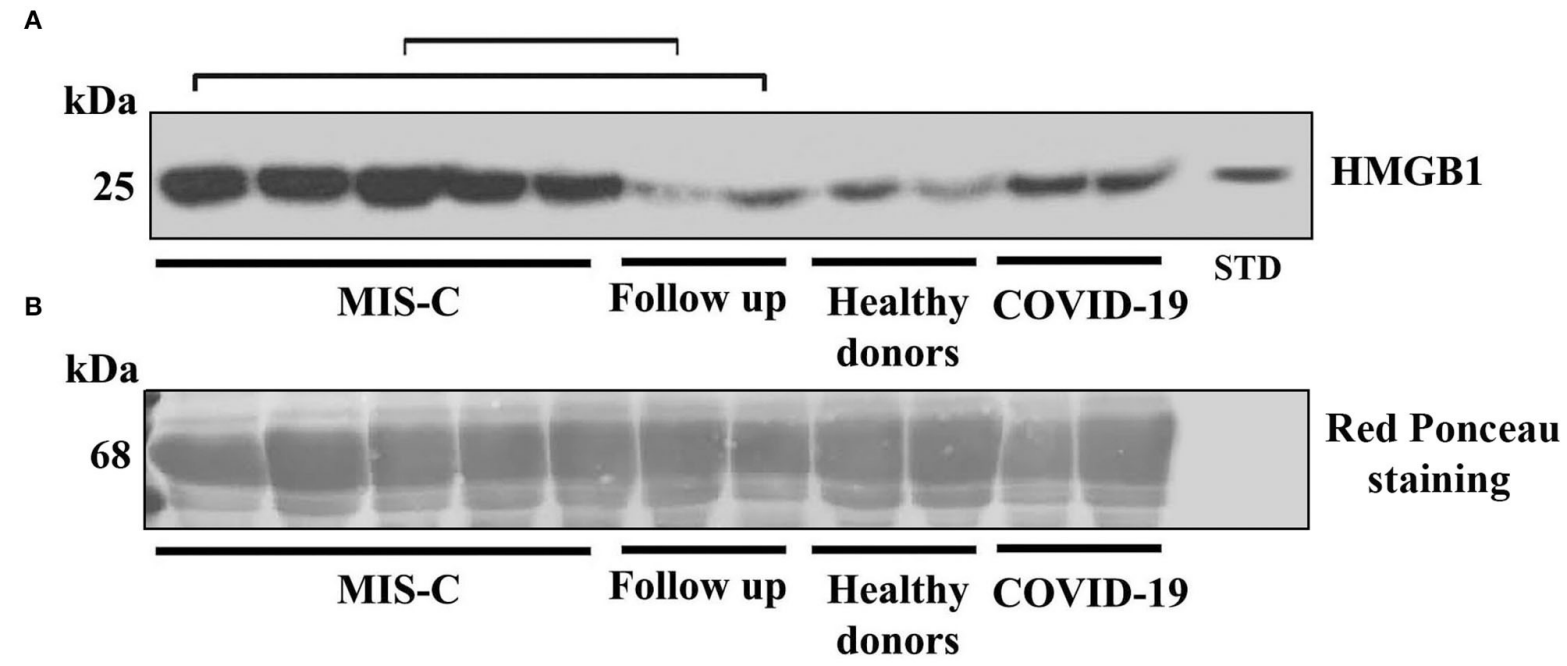
**(A)** Western blot analysis of high-mobility group box 1 (HMGB1) expression in the serum of patients with the multisystem inflammatory syndrome in children (MIS-C), children with a recent history of COVID-19, healthy donors, and in two of the five children that performed a follow-up as indicated by square brackets. A representative blot for each group is shown. Patients with MIS-C (*n* = 5), children with recent history of COVID-19 (*n* = 2), healthy donors (*n* = 2), and follow up (*n* = 2). HMGB1 levels were detected by Western blot using rabbit anti-HMGB1 polyclonal Ab, and proteins were detected using ECL reagents. HMGB1 standard was included in the blot as an internal control. **(B)** Blot membrane was stained for total protein with Red ponceau, as a control for loading and transfer. The kDa indicates the approximate region of the blot where albumin would be expected.

In this investigation, we tested HMGB1 expression by Western blot in sera from children with MIS-C, compared with children post COVID-19 and healthy donors. The results showed an evidently more increased expression of the HMGB1 band in patients with MIS-C, compared both with post patients with COVID-19 and controls (Figure 1).

Densitometric analysis confirmed that the median level of HMGB1 in the serum was significantly different in the three groups (MIS-C: 1,151.38 densitometric units (DU) vs. post COVID-19: 545.90 DU and vs. control: 234.04 DU, *p*-value < 0.0001 (using the Kruskal–Wallis test).

**Figure 2:**
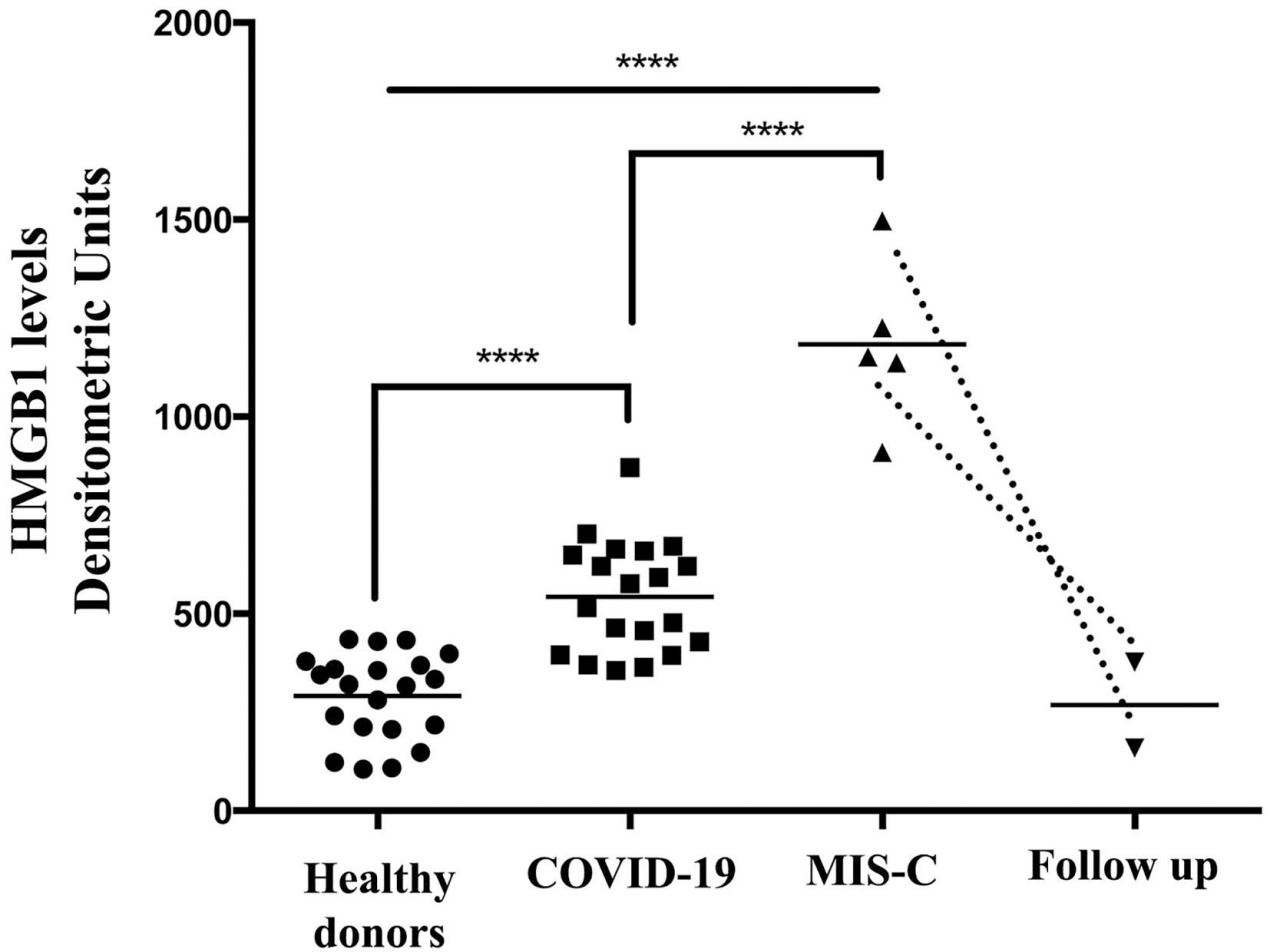
High-mobility group box 1 levels were detected by Western blot in the sera of patients with MIS-C (*n* = 5), children with a recent history of COVID-19 *(n* = 20), healthy donors (*n* = 21), and in two of the five children that performed a follow-up. Densitometric values of HMGB1 levels are represented and summarized by Scatter plot analysis. Serum HMGB1 levels from both MIS-C children and pediatric subjects with a recent history of COVID-19 were compared to healthy donors. ****p < 0.0001.

Thus, the median level of HMGB1 in children with MIS-C was significantly higher than in patients with post-COVID-19 and controls (Figure 2). Interestingly, in two of the five children that performed a follow-up, the HMGB1 value 40 and 52 days after the treatment decreased to levels that were superimposable to the ones of the control group. We found no correlation between HMGB1 and C-RP levels in this group (*p* = 0.624).

When we compared the HMGB1 level in patients with MIS-C, we found that patients with coronary involvement had a slightly but not statistically significant higher value of HMGB1 with respect to patients without coronary dilation (1,225.36 DU vs. 1,030.49 DU, *p* = 0.248 using the Kruskal–Wallis test).

The median of HMGB1 was also significantly higher in the children with post-COVID-19 compared with controls (*p* < 0.0001) (Figure 2).

If children belonging to the post COVID-19 group are categorized into symptomatic (*n* = 10) and asymptomatic (*n* = 10) patients, based on the symptoms that they had during the SARS-CoV2 infection, we found no difference in the HMGB1 level (545.90 DU (range: 369.90–701.71) vs. 510.18 DU (range: 356.00 – 870.48), *p* = 0.450).

## Discussion

In our study, we evaluated the levels of HMGB1 protein in the sera of children with MIS-C, patients with a recent history of COVID-19, and healthy children. The median level in patients with MIS-C was significantly higher than control groups. The HMGB1 level was also found to be slightly elevated when we compared patients with a recent history of COVID-19 patients with healthy children.

The higher HMGB1 level in the serum of patients after SARS-CoV-2 infection with respect to control subjects suggests a possible link between HMGB1 and COVID-19. In particular, HMGB1, an important pro-inflammatory molecule, may play a crucial role in the pathogenesis of MIS-C. Since COVID-19 and MIS-C are both characterized by the severe inflammatory immune response (15), HMGB1 may represent an alarm signal, indicating an increase in pro-inflammatory triggers.

The role of SARS-CoV-2 infection in MIS-C has been extensively demonstrated in the literature (23, 24) as well as their temporal relationship (4, 5).

The clinical presentation of patients with MIS-C can vary, i.e., children may suffer from different symptoms involving different organ systems, some with a typical clinical picture similar to KD, with coronary artery lesions. Common to all these children are the evident laboratory inflammation and positive serology for anti-SARS-CoV-2 antibodies. We confirmed that children with MIS-C are characterized by high inflammatory markers and lymphopenia associated in some cases with thrombocytopenia, supporting the evidence that this feature is a hallmark of SARS-CoV2 complication (18, 24). In previous studies, it has also been demonstrated that children with MIS-C had specific T and B cell subsets in the peripheral blood. Consiglio et al. (22) found higher central memory and effector memory CD4^+^ T cells, with lower naïve CD4^+^ T cells, and Licciardi et al. (19) revealed a characteristic B cell subset in MIS-C patients with low levels of transitional B cells and high levels of switched memory B cells and marginal zone B cells.

The significantly higher level of HMGB1 in MIS-C compared with control groups confirms its role in the hyperinflammatory state of these patients. It seems that MIS-C can share with KD some inflammatory pathways, besides some clinical aspects, in particular the cardiac involvement. To the best of our knowledge, our study is the first one that evaluated the levels of HMGB1 in patients with MIS-C. Previous studies (12, 25) investigated the levels of HMGB1 in a group of patients with KD. Qian et al. (12) demonstrated higher levels of HMGB1 in patients with KD compared with healthy controls. In particular, they revealed that patients with coronary artery lesions had a significantly higher level of HMGB1 as compared with the group without coronary artery lesions. Moreover, using a rabbit model for KD, they were able to further demonstrate an increased expression of HMGB1 protein in the endothelial cells from rabbit coronary arteries, suggesting a role for HMGB1 in promoting coronary artery injury. Otherwise, in our study, we did not find significantly higher HMGB1 levels in patients with coronary dilation, probably due to the small sample size.

The role of HMGB1 in the pathogenesis of KD was also demonstrated in a study by Hoshina T et al. (25), showing significantly higher levels of HMGB1 in the sera of patients with KD compared with healthy controls (*p* < 0.001) and no significant differences with sepsis patients. Thus, they suggested HMGB1 as a marker of systemic inflammation in KD and as having a possible role in mediating immune response in KD as in sepsis.

After specific therapy and clinical recovery, the HMGB1 levels of the two patients with MIS-C that performed a follow-up significantly decreased and were comparable to the values of the healthy control group. It confirms and extends the results of Hoshina et al. (25) and Quian et al. (12), who observed a gradual decrease of HMGB1 in the subacute and convalescent phase of KD.

Beyond similarities, recent studies reported huge differences in the cytokine profiles of patients with MIS-C, severe COVID-19 infection in adults, and KD (22), even if HMGB1 levels were not analyzed. In particular, adults with severe acute COVID-19 inflammation had higher levels of IL-7 and IL-8 than pediatric patients with KD and MIS-C, while IL-17A and endothelial and smooth muscle cell-derived neuropilin-like (ESDN) were higher in patients with KD than in patients with MIS-C (22).

Based on the previous evidence, together with our results, we suggested that HMGB1 may play an important role in the mechanism promoting the systemic clinical features of patients with MIS-C and possibly also be involved in the cardiac involvement and coronary dilation of patients with MIS-C.

When we considered the HMGB1 levels in children that had SARS-CoV2 infection 1 month prior, without any long-term complication, we found higher levels of HMGB1 than healthy controls, even in the absence of clear symptoms during the acute phase of infection. This result is quite surprising, and albeit two reports evaluating the level of HMGB1 in patients with COVID-19 (8, 9), during the acute phase, demonstrated that this protein was significantly higher in adults with severe acute COVID-19 disease. In the first study by Chen et al. (9), the patients admitted to the intensive care unit had a significantly higher level of HMGB1 with respect to patients hospitalized in the general ward, and in particular, in patients with fatal outcomes compared with alive patients. HMGB1 was also found to be positively correlated with high CT score and oxygen demand, indicating the severity of acute lung injury and ARDS. However, conversely to our result, elevated HMGB1 levels did not statistically differ in patients with COVID-19 hospitalized in the general ward with respect to healthy controls. In the subsequent study by Chen et al. (8), among COVID-19 hospitalized patients, the HMGB1 level was significantly higher in the severe group compared with the non-severe group. In this study, we did not find a correlation between the clinical presentation of patients with COVID-19 (symptomatic vs. asymptomatic) and HMGB1 levels. However, none of our patients has been hospitalized during the acute phase of COVID-19, and only half of the pediatric patients presented with mild COVID-19 disease. The difference with the data reported in the literature could be due to the age difference in the population under test and to the different clinical presentations. The difference in the immune response can be the reason for serious illness in adults compared with children (25).

We can suppose that SARS-CoV2 infection in children can determine a hyperinflammatory state, without necessarily leading to a serious clinical picture. It has also been demonstrated that high levels of HMGB1 can decrease the expression of the ACE2 receptor through HMGB1-RAGE interaction (8). One of the reasons why children seem to have a lower risk of infection and milder forms of the disease is that they express lower numbers of the ACE2 receptor (26) in their nasal and lung epithelium (27, 28). The association between HMGB1 levels and the expression of the ACE2 receptor in patients with COVID-19 has not been studied yet, so in future studies, it would be interesting to evaluate their possible correlation, as a mechanism that can modulate the entry of the virus into the respiratory cell.

Limitations of our study are that it is a single-center study with a small sample size of patients with MIS-C, which is, however, an extremely rare long-term complication of pediatric SARS-CoV2 infection. Another limitation is that we had only one patient with a clinical manifestation that required PICU admission, and the other four cases presented with less severe MIS-C could be handled by a general ward.

In conclusion, this is the first study, to the best of our knowledge, reporting the levels of HMGB1 in a group of pediatric patients shortly after the SARS-CoV-2 infection. The significant high level of HMGB1 protein found in the serum of patients with COVID-19 and MIS-C confirms its role in driving inflammation and highlights its involvement in their pathogenesis and severe manifestation. Although HMGB1 had been seen as an inflammatory marker in several pathological conditions, from a practical point of view, it may represent a useful tool for monitoring patients with MIS-C and their response to therapy, even if the possibility to use HMGB1 in routinely clinical monitoring is partially limited by the availability of this testing so far. This molecule could be targeted by specific therapies, since monoclonal antibodies against HMGB1 demonstrated to be effective in refractory epilepsy (29) and glycyrrhizin, an additional HMGB1 inhibitor, was able to protect from neuronal inflammation in a mouse model of autoimmune encephalomyelitis (30). Thus, further research is warranted to improve our understanding of HMGB1 as a potential biomarker and therapeutic target in patients with severe illness.

## Data Availability Statement

The raw data supporting the conclusions of this article will be made available by the authors, without undue reservation.

## Ethics Statement

The studies involving human participants were reviewed and approved by The Research and Ethics Committee of the Policlinico Umberto I Hospital approved the study (n. 0399/2021). Written informed consent to participate in this study was provided by the participants' legal guardian/next of kin.

## Author Contributions

LP and VM wrote the first draft of the manuscript. LP, VM, and RN performed the statistical analysis. AF and SB organized the database. EM and TG wrote sections of the manuscript. MS, RM, and FM supervised the work and contributed to conception and design of the study. All authors contributed to manuscript revision, read, and approved the submitted version.

## Conflict of Interest

The authors declare that the research was conducted in the absence of any commercial or financial relationships that could be construed as a potential conflict of interest.

## Publisher's Note

All claims expressed in this article are solely those of the authors and do not necessarily represent those of their affiliated organizations, or those of the publisher, the editors and the reviewers. Any product that may be evaluated in this article, or claim that may be made by its manufacturer, is not guaranteed or endorsed by the publisher.

## Abbreviations

COVID-19: coronavirus disease 2019
SARS-CoV2: severe acute respiratory syndrome coronavirus 2
MIS-C: multisystem inflammatory syndrome in children
HMGB1: high-mobility group box 1
KD: Kawasaki disease
DAMPs: damage-associated molecular patterns
TLRs: activating toll-like receptors
RAGE: advanced glycosylation end products
IVIG: intravenous immunoglobulin
CLIA: chemiluminescence immunoassay.
